# Mid-term Results of Core Decompression Combined With Bone Marrow Aspirate Concentrate (BMAC) Injection for Early Osteonecrosis of the Femoral Head: A Prospective, Pilot Study

**DOI:** 10.7759/cureus.94788

**Published:** 2025-10-17

**Authors:** Sunil Hegde, Mohammed Schezan Iqbal, Rajiv Kaul, Hrishikesh Pande

**Affiliations:** 1 Department of Orthopaedics, Military Hospital Khadki, Pune, IND; 2 Department of Orthopaedics, Indian Naval Hospital Ship Asvini, Mumbai, IND; 3 Department of Orthopaedics, Base Hospital Delhi Cantonment, Delhi, IND; 4 Department of Sports Medicine, Armed Forces Medical College, Pune, IND

**Keywords:** avascular osteonecrosis, bone marrow aspirate concentrate (bmac), hip preservation, mesenchymal stem cells (mscs), regenerative orthopaedics

## Abstract

Introduction: Osteonecrosis of the femoral head (ONFH) is a chronic, debilitating affliction of the hip, with a high incidence in the employable population, thus posing a significant social and economic burden. To date, total hip arthroplasty (THA) remains the only successful treatment for the late stages of ONFH. Various joint-preserving surgical options have been described for early ONFH, with varying success rates. The study aimed to analyze the functional and radiological outcomes of core decompression (CD) combined with bone marrow aspirate concentrate (BMAC) injection for early ONFH.

Material and methods: A total of 30 patients with ONFH, Ficat-Arlet Stage I, IIA, and IIB, underwent CD with BMAC injection under fluoroscopic guidance, followed by a period of protected weight-bearing and physiotherapy. Patients were reviewed at 3, 9, and 15 months to look for improvement in the following parameters: pain on visual analogue scale (VAS), hip range of motion (ROM), weekly analgesic requirement, Harris Hip Score (HHS), and reduction in size of lesion on magnetic resonance imaging (MRI).

Results: The mean age of the study population was 41.2 ± 6.74 years. Mean VAS scores and HHS significantly improved over time (Friedman's test, p < 0.001). Median weekly analgesic doses reduced from 14 (interquartile range (IQR): 10-18) preoperatively to 5 (IQR: 3-7) at 3 months, 3 (IQR: 1-5) at 9 months, and 2 (IQR: 0-4) at 15 months. Following the intervention, hip abduction and external rotation improved significantly (Friedman's test, p < 0.01). While MRI analysis showed an initial significant reduction in size of the lesion at 3 months (p < 0.01, r = 0.33), subsequent follow-ups showed a significant increase (p < 0.01 at 9 months, r = 0.35; p < 0.05 at 15 months, r = 0.29), suggesting limited durability of radiological improvement or possible disease progression despite initial response. A Kaplan-Meier survival analysis indicated a 30% progression rate to Stage III.

Conclusion: While CD with BMAC injection has definite advantages in decreasing pain and improving hip ROM, our results suggest that patients with smaller initial lesions and better baseline function may have a more favorable outcome than those with more widespread involvement of the weight-bearing dome. However, MRI findings and a survival analysis indicated a 30% progression rate to Stage III, raising concerns about the long-term efficacy of this treatment.

## Introduction

Osteonecrosis of the femoral head (ONFH) is a progressive, debilitating disorder that may lead to hip osteoarthritis (OA), resulting in prolonged morbidity and increased healthcare costs. Its observed incidence in the UK population during 1989-2003 was between 1.4 and 3.0 per 1,00,000 individuals [1]. Currently, the accepted pathophysiological mechanism is presumed to arise from an interaction between altered bone physiology, vascular impairment, genetic predisposition, and identifiable risk factors such as alcohol consumption, corticosteroid administration, trauma, hypercoagulable conditions such as sickle-cell anaemia, and decompression sickness [2]. However, most cases are idiopathic, with no identifiable cause or risk factor [3]. Treatment begins conservatively with physical therapy and medications such as bisphosphonates and statins, and many patients benefit symptomatically in the short term [4]. A substantial number of cases progress to painful hip OA, which necessitates surgical interventions in the form of either joint-preserving procedures or total hip arthroplasty (THA).

## Materials and methods

This prospective pilot study evaluates the effects of core decompression (CD) with autologous bone marrow aspirate concentrate (BMAC) injection for early stages of ONFH. The study was conducted at a tertiary care hospital from January 2015 to December 2017. The Institutional Ethics Committee clearance (no IEC/2015/104 dated 08/10/2014) was obtained before the start of the study. Our study included individuals aged >18 years with painful hips and radiographic features of ONFH, Ficat-Arlet Stage I, IIA, and IIB. Exclusion criteria included pregnancy, infective hip conditions, malignancy, patients on corticosteroid medication, and any previous surgical procedure for ONFH. As this was a pilot study, the sample size for our study hypothesis was estimated to be 30 hips. All eligible subjects were evaluated preoperatively with history and clinical examination, followed by plain radiography (standard anteroposterior and frog-leg lateral views) and magnetic resonance imaging (MRI) of both hips (Siemens; Magnetom Symphony®, 1.5T). MRI was performed using a variety of imaging sequences (T1-weighted, T2-weighted, and short tau inversion recovery images) in which a single, independent radiologist reported the results. Baseline parameters included associated comorbidities, details of alcohol consumption, pain on visual analogue scale (VAS), hip range of motion (ROM), weekly analgesic requirement, Harris Hip Score (HHS), and Ficat-Arlet stage of the disease. We hypothesised that CD + BMAC improves pain and hip function and delays radiological progression compared to baseline.

Surgical technique

**Figure 1:**
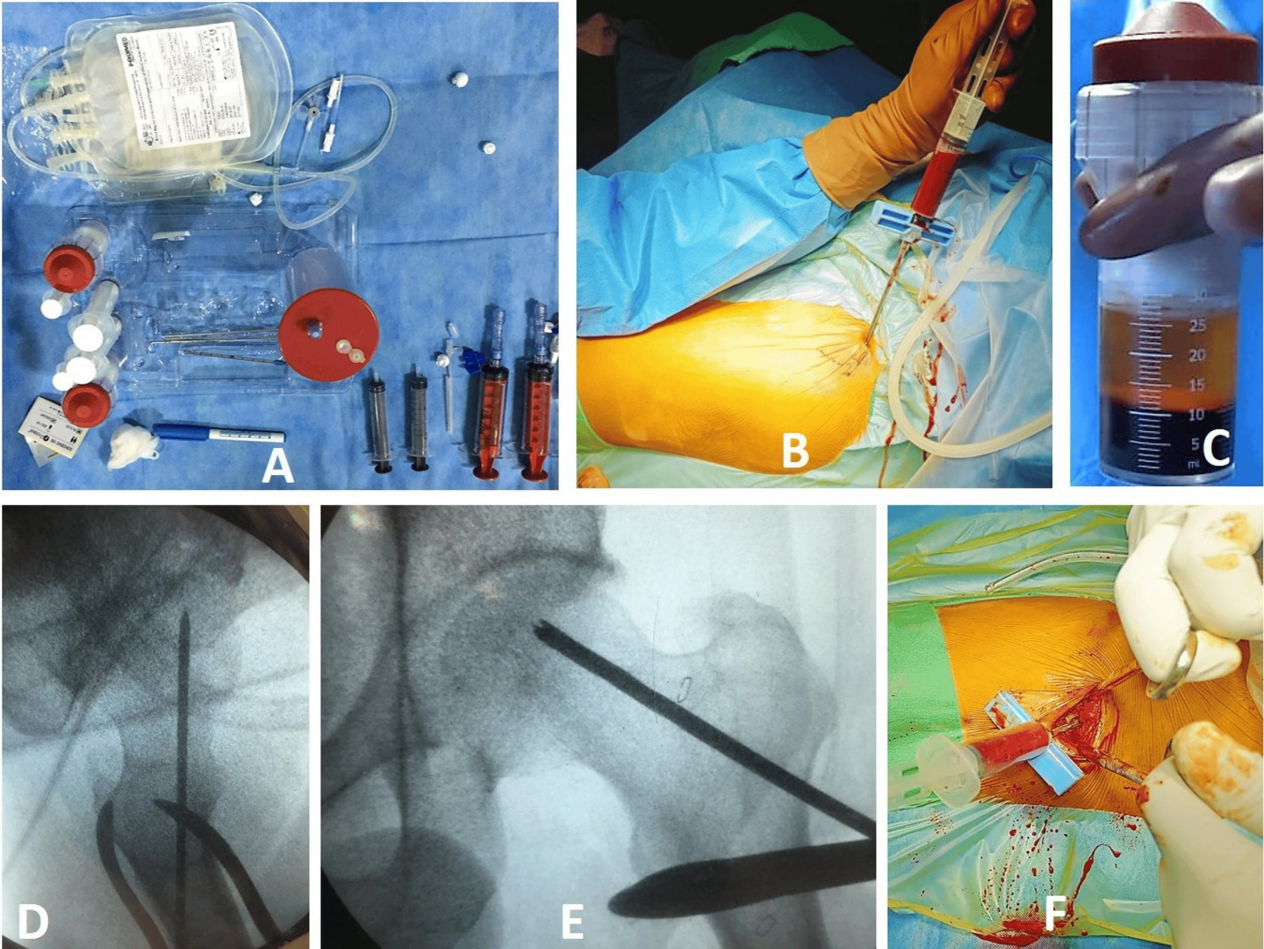
1A: Components of a commercially available BMAC kit (Concemo®, Soluciones Bioregenerativas SL, Gava, Spain). 1B: Bone marrow aspiration from ipsilateral iliac crest; multiple sites are chosen to get maximum harvest of mesenchymal stem cells. 1C: 10 mL of BMAC obtained after centrifugation. 1D and E: Insertion and drilling over a guidewire directed toward the necrotic focus/cyst under fluoroscopic guidance. 1F: Injection of BMAC through previously created track using the same bone marrow needle, with caution to prevent backflow of contents BMAC: Bone marrow aspirate concentrate

Under spinal or epidural anesthesia, patients were positioned on a fracture table for fluoroscopic imaging. A 30 mL VacLok® syringe (Merit OEM, Utah, USA), flushed with heparin in a concentration of 5000 U/mL, was used to aspirate bone marrow from the ipsilateral iliac crest (Figure 1B). Aspiration was performed using a sterile, disposable bone marrow needle for 5-10 mL from multiple sites, to a total of 60 mL of bone marrow, which was then pooled and evenly mixed with 5 mL of anticoagulant in a blood collection bag (Figure 1A). The mixture was transferred to the centrifugation containers of a commercially available kit (Concemo®, Soluciones Bioregenerativas SL, Gava, Spain) to prepare the concentrated bone marrow (Figure 1A). The mixture was centrifuged for 13 minutes at 20-25˚C to separate the blood constituents, from which 10 mL of bone marrow concentrate was obtained (Figure 1C). Next, femoral head CD was performed under fluoroscopic guidance (Figures 1D, 1E). Using a guidewire and 4.5 mm cannulated drill bit, 2-3 drill holes were made into the necrotic focus through a single entrance drill hole in the lateral cortex, following which BMAC was injected using the sterile needle (Figure 1F), with thumb pressure for a minute over the entry site, to avoid backflow of contents.

Postoperative protocol included anticoagulation with oral aspirin (150 mg) for three weeks and non-weight-bearing, walker-assisted ambulation for four weeks, with progression to crutches, and ultimately full, unassisted weight bearing at the end of three months. High-impact physical activities such as running and sports were not recommended for six months. Follow-up visits were performed every three months for up to 15 months after surgery. Functional parameters such as hip ROM, VAS scores, HHS, analgesia requirement, and radiographs of both hips were assessed at periodic intervals up to 15 months postoperatively. MRIs were obtained at 3, 9, and 15 months to look for revascularization of the femoral head and resolution of lesions.

## Results

Of the 30 patients, 23 were males (76.7%) while seven were females (23.3%). The mean age of the study population was 41.2 ± 6.74 years. Based on the subjects' history of comorbidities, we had 26 patients without any known comorbidity and four patients who had type II diabetes mellitus, ischaemic heart disease, old pulmonary tuberculosis, and hypertension, respectively. Only 10 (33.3%) of the subjects gave a history of moderate alcohol consumption, while 20 (66.7%) did not. The average follow-up period was 16.3 (±0.2) months, with the most extended follow-up being 2.5 years. The various preoperative variables documented are represented in Table 1.

**Table 1:** Demographic and preoperative variables VAS: Visual analogue scale

Total number (n)	30
Age	Mean: 41.2 ± 6.74 years
	Range: 24-67
	SD: 6.74
Sex	Female: 7 (23.3%)
	Male: 23 (76.7%)
Side	Right: 16 (53.3%)
	Left: 14 (46.7%)
BMI	Mean: 25.5 kg/m^2^
	Range: 20-32
	SD: 3.07
Alcohol intake	Yes: 10 (33.33%)
	No: 20 (66.66%)
Pre-op VAS score	Mean: 6.3
	Range: 3-9
	SD: 2.04
Pre-op Harris Hip Score	Mean: 75.03
	Range: 55-82
	SD: 4.86
Thigh wasting on the affected side	Mean: 1.68 cm
	Range: 1-4 cm
	SD: 0.55
Ficat-Arlet staging	Stage I: 9 (30%)
	Stage II: 21 (70%)
Range of motion of the affected hip	Mean flexion: 102° (range 90°-110°)
	Mean abduction: 33.3° (range 20°-50°)
	Mean adduction: 25.6° (range 15°-30°)
	Mean internal rotation: 4.83° (range 0°-20°)
	Mean external rotation: 30° (range 20°-40°)
Duration of surgery	Mean: 65.3 minutes
	Range: 58-74 minutes

Pain and functional outcomes

**Figure 2:**
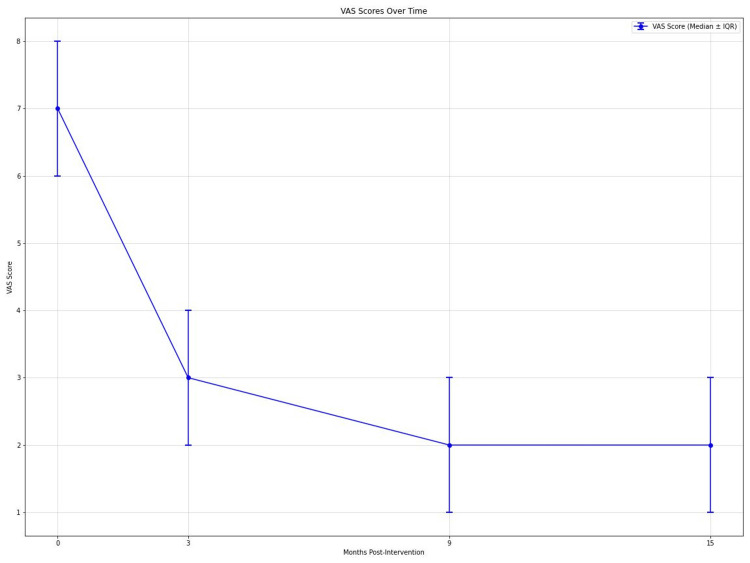
Graph showing declining VAS scores over time at 0, 3, 9, and 15 months postintervention. VAS: Visual analogue scale

**Figure 3:**
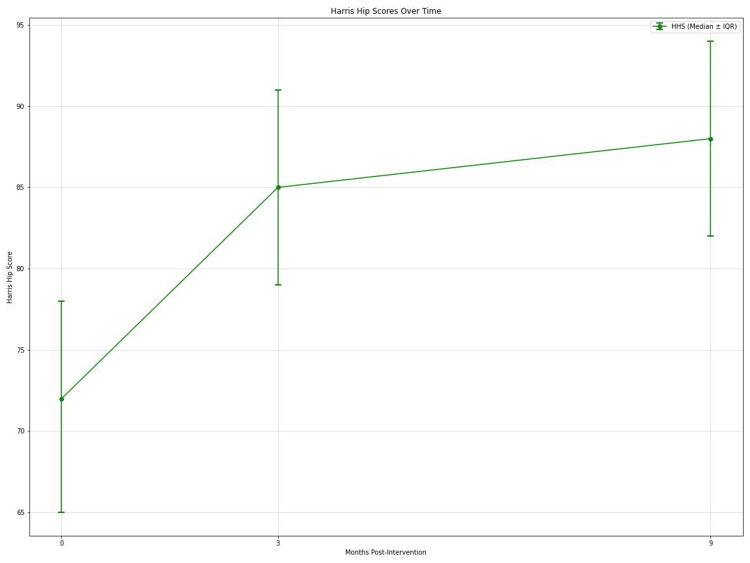
Graph depicting HHS over time at 0, 3, and 9 months postintervention. HHS: Harris Hip Score

VAS scores for pain showed significant improvement over time (Friedman's test, p < 0.001). Median VAS scores decreased from 7 (interquartile range (IQR): 6-8) preoperatively to 3 (IQR: 2-4) at 3 months, 2 (IQR: 1-3) at 9 months, and 2 (IQR: 1-3) at 15 months postintervention (Figure 2). Post hoc analysis revealed significant differences between preoperative scores and all follow-up time points (all p < 0.001), with large effect sizes (r = 0.62, 0.65, and 0.67 at 3, 9, and 15 months, respectively). HHS also improved significantly (Friedman's test, p < 0.001). Median HHS increased from 72 (IQR: 65-78) preoperatively to 85 (IQR: 79-91) at 3 months and 88 (IQR: 82-94) at 9 months (Figure 3). Significant differences were observed between preoperative and 3-month scores (p < 0.001, r = 0.58), preoperative and 9-month scores (p < 0.001, r = 0.61), and 3-month and 9-month scores (p = 0.03, r = 0.28). Analgesic requirements decreased significantly over the study period (Friedman's test, p < 0.001). Median weekly analgesic doses reduced from 14 (IQR: 10-18) preoperatively to 5 (IQR: 3-7) at 3 months, 3 (IQR: 1-5) at 9 months, and 2 (IQR: 0-4) at 15 months. All post hoc comparisons with preoperative values were significant (all p < 0.001) with large effect sizes (r = 0.59, 0.63, and 0.64 at 3, 9, and 15 months, respectively).

ROM

**Figure 4:**
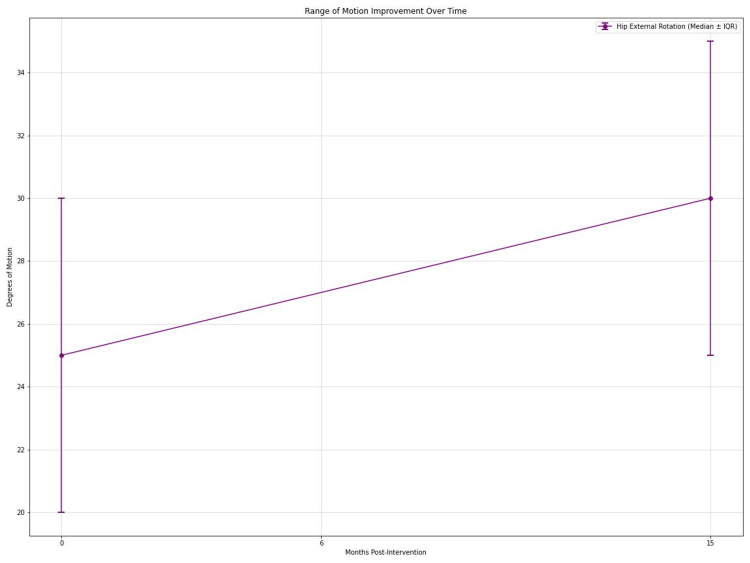
Graph showing improvement in mean hip external rotation in degrees over time at 0, 6, and 15 months.

Hip abduction improved significantly (Friedman's test, p < 0.001). Median abduction increased from 30° (IQR: 25°-35°) preoperatively to 35° (IQR: 30-40°) at 6 months and 38° (IQR: 33-43°) at 15 months. Post hoc analysis showed significant improvements at 6 months (p < 0.01, r = 0.34) and 15 months (p < 0.001, r = 0.42) compared to preoperative values. External rotation also improved significantly (Friedman's test, p < 0.01). Median external rotation increased from 25° (IQR: 20°-30°) preoperatively to 30° (IQR: 25°-35°) at 15 months (p < 0.01, r = 0.36) (Figure 4).

MRI lesion size

**Figure 5:**
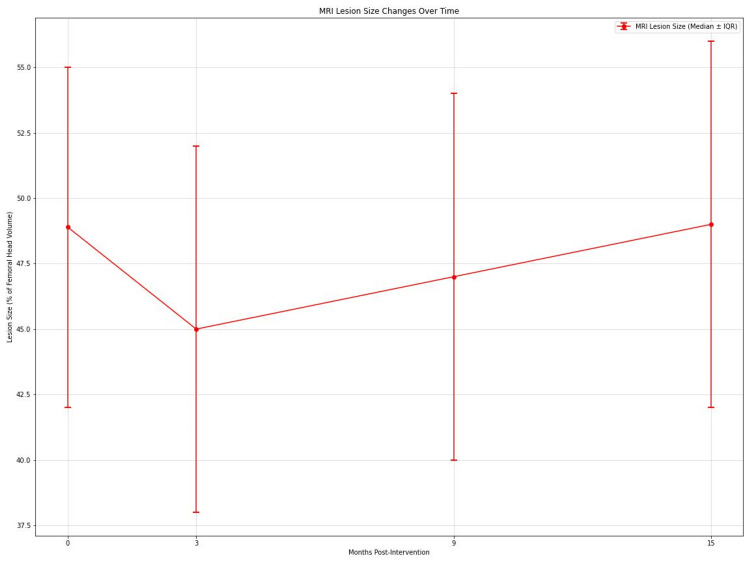
Graph depicting variation in size of lesion (as percentage of femoral head volume) on MRI over time at 0, 3, 9, and 15 months postintervention.

MRI lesion size, expressed as a percentage of femoral head volume, showed significant changes over time (Friedman's test, p < 0.001). Median lesion size decreased from 48.9% (IQR: 42%-55%) preoperatively to 45% (IQR: 38%-52%) at 3 months, then increased to 47% (IQR: 40-54%) at 9 months and 49% (IQR: 42-56%) at 15 months (Figure 5). Post hoc analysis revealed significant differences between preoperative and 3-month values (p < 0.01, r = 0.33), 3-month and 9-month values (p < 0.01, r = 0.35), and 9-month and 15-month values (p < 0.05, r = 0.29).

Disease progression

**Figure 6:**
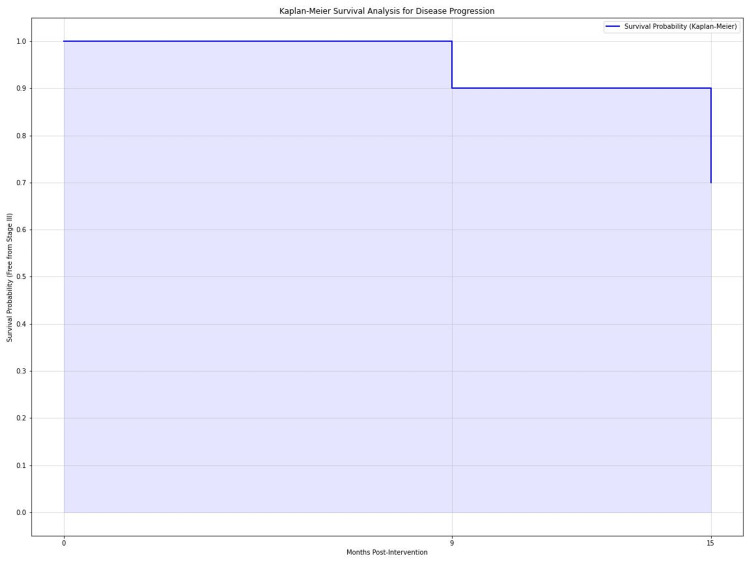
Kaplan-Meier survival analysis for disease progression depicting the probability of hip survival (free from Stage III disease) at 0, 9, and 15 months postintervention.

Kaplan-Meier survival analysis estimated the probability of remaining free from progression to Ficat-Arlet Stage III as 90% (95% CI: 84-96%) at 9 months and 70% (95% CI: 62-78%) at 15 months (Figure 6) for both Stage I and Stage II lesions. The cumulative incidence of Stage III progression was 10% (95% CI: 4-16%) at 9 months and 30% (95% CI: 22-38%) at 15 months. Multivariate analysis using logistic regression identified initial lesion size (OR = 1.08, 95% CI: 1.02-1.14, p = 0.01) and baseline HHS (OR = 0.95, 95% CI: 0.91-0.99, p = 0.02) as significant predictors of disease progression to Stage III. Age was not a significant predictor (OR = 1.05, 95% CI: 0.98-1.12, p = 0.15). A comparison of all outcome variables (preoperative versus postoperative at 3, 9, and 15 months) is summarised in Table 2.

**Table 2:** Comparison of all outcome variables (preoperative versus postoperative at 3, 9, and 15 months) The statistical tests used were the Friedman's test for deriving the p-values of all variables. VAS: Visual analogue scale

Variable	Pre-op	3 months		p-value (for change at 3 months compared with pre-op value)	9 months		p-value (for change at 9 months compared with pre-op value)	15 months		p-value (for change at 15 months compared with pre-op value)
Analgesic requirement	4.5	0.9		<0.001	1.6		<0.001	1.6		<0.001
Mean (tablets/week)	2 to 9	0 to 4			0 to 8			0 to 9		
SD	1.2	1.24			1.4			1.5		
VAS (median)	6.3	2.66		<0.001	3.13		<0.001	3.8		<0.001
SD	2.04	1.7			1.83			2.29		
Harris Hip Score (mean)	75.27	80.97		<0.001	75.27		0.014	75.43		0.936
SD	11.46	11.01			11.46			19.29		
Ficat-Arlet staging	Stage I: 9 (30%)	Stage I: 9 (30%)		-	Stage I: 9 (30%)		-	Stage I: 9 (30%)		-
	Stage II: 21 (70%)	Stage II: 21 (70%)			Stage II: 18 (60%)			Stage II: 12 (40%)		
					Stage III: 3 (10%)			Stage III: 9 (30%)		
Size of lesion on MRI (mean) as % of femoral head	31.67	29.33		0.032	47.50		0.04	47.20		0.041
SD	7.47	11.12			15.5			15.4		
Flexion (in degrees) (mean)	102	102.3		0.662	101		0.375	97.33		0.225
SD	7.14	7.74			7.59			19.99		
Adduction (in degrees) (mean)	25.17	25.5		0.326	24.67		0.415	23.33		0.102
SD	5.33	5.31			6.81			8.44		
Abduction (in degrees) (mean)	33.3	33.3		1	30		0.023	26.67		0.003
SD	8.02	8.02			12.36			16.26		
Internal rotation (in degrees) (mean)	4.83	4.5		0.326	5		0.712	5		0.712
SD	5.17	4.42			4.55			4.55		
External rotation (in degrees) (mean)	31.67	31.67		0.999	31		0.326	29.33		0.032
SD	7.47	7.91			8.45			11.12		
Wasting (in cm) (mean)	1.6	1.6		-	1.2		<0.001	1.1		<0.001
SD	0.165	0.165			0.925			1.03		

## Discussion

Ficat described CD as a means for treating precollapse lesions by reducing the intra-osseous pressures and increasing the femoral head vascular inflow [5]. He also reported having obtained good results in 90% of all subjects with Stage I and II disease in 133 hips. Following a few studies that were promising enough, and with the possibility of using adjuvant materials in the canals created during the process of CD, Gangji et al. published a study of 24 hips of Stages I and II, which were randomised into a treatment of CD alone or CD associated with bone marrow injection [6]. They concluded that a longer time was needed to see the features of osseous collapse in the CD group associated with bone marrow injection. The definite recommendations were that CD is more cost-effective than observation alone, and it should be the first-line treatment for hips with early stages of ONFH [7].

Cell therapy has been advocated as a therapeutic resource that aids bone formation and remodelling in the early, precollapse stages of osteonecrosis. Consequently, the supplementation of multipotent stem cells (MSCs) to an established technique of CD has been developed, due to their capacity to retain mitotic multiplication, while also being able to differentiate into different cell lineages, such as adipocytes, chondrocytes, osteoblasts, and osteocytes. We based our study on the presumption that these stem cells would repopulate the bony trabeculae of the femoral head's necrotic section and enhance regeneration and remodelling of the necrotic focus [8]. Our study investigated the efficacy of autologous BMAC injection following CD in managing early-stage ONFH. The results demonstrate significant improvements in pain and function but also reveal limitations in halting disease progression.

Pain and functional outcomes

The statistically significant reduction in VAS scores for pain at all follow-up points (p < 0.001) with large effect sizes (r = 0.62, 0.65, and 0.67 at 3, 9, and 15 months, respectively) indicates substantial pain relief following the procedure. This improvement aligns with findings from previous studies [9]. The reduction in pain is likely due to decreased intra-osseous pressure and improved vascularisation resulting from CD, combined with the regenerative effects of bone marrow concentrate [10]. HHS showed significant improvement at 3 and 9 months (p < 0.001), with effect sizes of r = 0.58 and r = 0.61, respectively. This functional improvement corresponds with the pain reduction and is consistent with other studies reporting enhanced functional outcomes using BMAC [11]. The significant decrease in analgesic requirements throughout the follow-up period (p < 0.001) further supports the procedure's efficacy in pain management. This reduction in medication use could potentially prevent complications associated with long-term analgesic use [12].

ROM and physical findings

Improvements in hip abduction and external rotation were statistically significant at later follow-ups (6 and 15 months), suggesting a gradual functional recovery. However, the lack of significant improvement in other ranges of motion indicates that the procedure may have limitations in restoring full hip mobility.

Radiological outcomes and disease progression

The MRI findings present a complex picture. While there was an initial significant reduction in lesion size at 3 months (p < 0.01, r = 0.33), subsequent follow-ups showed substantial increases (p < 0.01 at 9 months, r = 0.35; p < 0.05 at 15 months, r = 0.29). This pattern suggests that while the procedure may improve initially, it may not consistently prevent disease progression [13]. Kaplan-Meier survival analysis revealed that 30% of hips progressed to Ficat-Arlet Stage III by 15 months. This progression rate is concerning and indicates that the procedure may not alter the natural course of the disease in all cases. Our findings are somewhat consistent with Gangji et al., who reported disease progression in 23.08% of treated hips at 5 years, although our progression rate appears higher in a shorter timeframe [14].

Predictors of outcome

The multivariate analysis identified initial lesion size (OR = 1.08, 95% CI: 1.02-1.14, p = 0.01) and baseline HHS (OR = 0.95, 95% CI: 0.91-0.99, p = 0.02) as significant predictors of disease progression. This outlines the importance of early intervention and suggests that patients with smaller initial lesions and better baseline function may have more favourable outcomes [15].

Limitations and future directions

Our study has several limitations. The sample size of 30 hips, while sufficient for a pilot study, limits the external validity of our findings. The lack of a control group prevents direct comparison with CD alone or non-operative measures, so comparative efficacy cannot be conclusively drawn [16]. Due to technical constraints, we could not quantify the exact number of bone marrow-derived MSCs (BM-MSCs) used in each case [17]. The follow-up period of 15 months, while providing valuable mid-term data, may not be sufficient to assess long-term outcomes. Further studies, specifically randomised controlled trials, with longer follow-up periods, are necessary to understand the procedure's impact on disease progression and hip survival in order to prevent subsequent conversions to THA [18].

## Conclusions

Our study demonstrates that CD with autologous BMAC injection provides significant mid-term improvements in pain and function for patients with early-stage ONFH. However, the radiological findings and disease progression rates suggest that the procedure may not consistently alter the natural course of the disease. In the absence of a control group, the comparative efficacy of the intervention cannot be conclusively drawn, and further multicenter trials are required to validate the findings. While this technique is promising and could surpass CD alone, further studies on the cellular-level reparative mechanisms are needed. Future research should also aim to optimize the quantity and quality of BM-MSCs used, as this may be crucial for improving outcomes and potentially slowing disease progression.
